# P038: Efficacy of prevention measures against nosocomial influenza at a large university hospital

**DOI:** 10.1186/2047-2994-2-S1-P38

**Published:** 2013-06-20

**Authors:** A Iten, T Bouvard, Y Thomas, C Bonfillon, C Ginet, L Kaiser, C-A Siegrist, D Pittet

**Affiliations:** 1 Infection Control Program, University of Geneva Hospitals (HUG), Geneva, Switzerland; 2 Department of Health Employees, University of Geneva Hospitals (HUG), Geneva, Switzerland; 3 Laboratory of Virology, University of Geneva Hospitals (HUG), Geneva, Switzerland; 4 Department of Child and Adolescent Medicine, University of Geneva Hospitals (HUG), Geneva, Switzerland

## Introduction

At HUG, healthcare workers (HCW) are currently obliged to be vaccinated or wear masks in ward corridors and patient rooms during seasonal influenza (SI) epidemics. Since winter 2011-12, HCWs vaccinated against SI wear a badge with the text "I am vaccinated to protect you" and, since winter 2012-13, those who are not vaccinated wear a badge with the text "I wear a mask to protect you". Regular audits have allowed to quantify compliance with recommendations in parallel with active recording of influenza cases.

## Methods

During the SI epidemic, an audit recorded HCWs with a badge and those with correct mask wear over 2 periods of 2 weeks each in Feb−March 2012 and 3 periods of 2 weeks each in Jan−March 2013. Compliance was assessed as follows: (number of HCWs wearing a colored badge + number of HCWs wearing a mask correctly)/number of HCWs observed = number of compliant HCWs/number of HCWs observed, expressed as a percentage. Suspected cases of SI were confirmed by positive realtime RT-PCR reaction. Cases were defined as nosocomial (NOSO) SI when symptoms occurred >72 h post-admission.

## Results

Of 1390 HCWs observed in winter 2012, 469 wore a badge or mask (estimated compliance, 33.5%). In winter 2013, 2070/2937 observed HCWs were compliant (70.5%). We recorded 84 NOSO SI /152 SI (55.2%) and 96 NOSO SI /267 SI (35.9%) cases during 2011-12 and 2012-13, respectively. Compliance with recommendations in internal medicine averaged 68.6% in 2012 and 72.2% in 2013. The proportion of NOSO SI cases remained stable (30.3% and 21.6%, respectively). At the geriatric hospital, compliance progressed from 58.6% to 72.2%, while the proportion of NOSO SI cases decreased from 84.9% to 63.3%, respectively. These measures prevented an estimated number of 115 NOSO SI cases at HUG in 2012-13, together with a reduced number of deaths among SI patients.

## Conclusion

Mandatory badge wear, continuous SI epidemic surveillance and availability of compliance rates with recommendations decrease the risk of NOSO SI and improve patient safety.

## Disclosure of interest

None declared

